# Implementation of Good Clinical Laboratory Practice in an Immunology Basic Research Laboratory: The KEMRI-Wellcome Trust Research Laboratories Experience

**DOI:** 10.1093/ajcp/aqy138

**Published:** 2018-10-18

**Authors:** Horace Gumba, Jennifer Musyoki, Moses Mosobo, Brett Lowe

**Affiliations:** 1 KEMRI-Wellcome Trust Research Programme, Kilifi, Kenya; 2 Centre for Tropical Medicine and Global Health, University of Oxford, Oxford, UK

**Keywords:** Good Clinical Laboratory Practice (GCLP), Quality assurance, Quality system

## Abstract

**Objectives:**

Good Clinical Laboratory Practice (GCLP) is a standard that ensures quality and reliability of research data by adopting the principles of Good Laboratory Practice and Good Clinical Practice. Even though implementing a quality system in a basic research laboratory is still a contentious issue, it ensures that the research data are accurate, valid, and reliable. GCLP implementation requires proper documented procedures and safety precautions to achieve this objective.

**Methods:**

This article describes the Kenya Medical Research Institute (KEMRI)–Wellcome Trust Research Laboratories experience in the implementation of GCLP guidelines in a laboratory conducting basic research.

**Results:**

The laboratory managed to implement GCLP elements that could be applied to a basic research laboratory, such as standard operating procedures, equipment management, laboratory analytical plans, organization, and personnel. The laboratory achieved GCLP accreditation in October 2015.

**Conclusions:**

The methodology, suggestions, and comments that arose from our experience in implementing GCLP guidelines can be used by other laboratories to develop a quality system using GCLP guidelines to support medical research conducted to ensure the research data are reliable and can be easily reconstructed in other research settings.

The KEMRI-Wellcome Trust Research Programme was established in 1989 by the collaboration between the Kenya Medical Research Institute (KEMRI) and the Wellcome Trust and the University of Oxford, with the goal of using research to achieve better health for all in Africa working with the local community as well as developing African scientific leadership. The KEMRI-Wellcome Trust Research Laboratories integrated epidemiological, social, laboratory, and clinical research in parallel with results feeding into local and international health policy. It consists of four main state-of-the-art laboratories: clinical trials laboratory (CTL), short-turnaround laboratory (STAT), microbiology laboratory, and immunology basic research laboratory.

The immunology basic research laboratory of the KEMRI-Wellcome Trust Research Laboratories conducts research with a focus on host immune responses to malaria and human immunodeficiency virus infections as well as the impact of malnutrition on immune responses. The research mainly performed in this laboratory involves experiments that help advance the scientific knowledge on malaria transmission with the aim of developing a protective vaccine. The work is being done by a group of scientists with various backgrounds in immunology together with a team of several PhD and master’s students as well as technical staff. Because of the numerous scientific work undertaken, several international publications and presentations to international scientific conferences have been produced. As such, the reputation at KEMRI-Wellcome Trust Research Laboratories has been held high, and it continues to attract several collaborators and funding organizations to foster medical research in the field of immunology.

In 2014, there was a growing interest from the funding organizations to ensure that the immunology research laboratory get accredited. The funding organizations wanted their research work to be conducted in compliance with GCLP standards. Thus, there was need to develop a quality system in the immunology laboratory using the GCLP guidelines with the aim of it getting accredited. GCLP standards provided the best platform to achieve this because other laboratories within the KEMRI-Wellcome Trust Research Laboratories, such as CTL, STAT, and microbiology laboratories, were already GCLP accredited by Qualogy Accreditation Scheme Ltd (Kettering, UK). This article describes our experience in implementing the GCLP in an immunology basic research laboratory within the KEMRI-Wellcome Trust Research Laboratories. The laboratory achieved a GCLP accreditation in 2015, as indicated in **Figure 1**.

**Figure 1:**
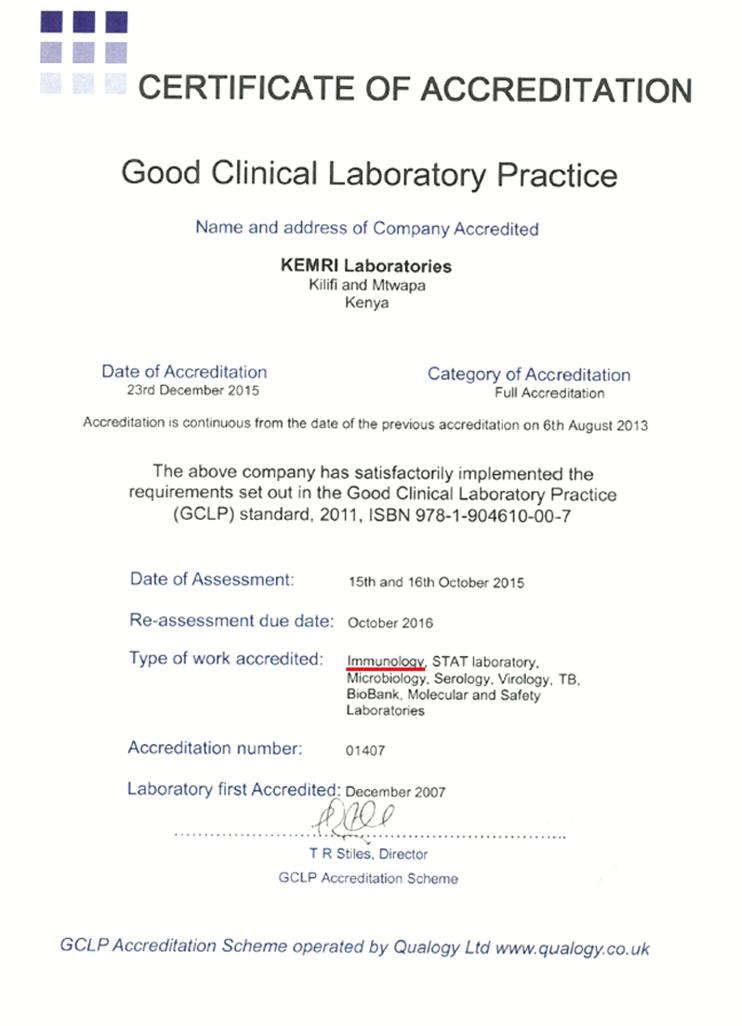
Good Clinical Laboratory Practice (GCLP) accreditation certificate.

## Objectives

The main objective of developing a quality system using GCLP standards was to provide research data that are of high quality and reliable while also achieving GCLP accreditation. GCLP was chosen as the best platform to achieve this because samples used for this research were being received from clinical trials. Furthermore, achieving GCLP accreditation would further boost our reputation and provide a better working relationship with our funders and sponsors. Achieving GCLP accreditation also helped us standardize some of the common procedures within a research setting such as equipment management, organization, and standard operating procedures.

## Personnel Organization and Training Records

**Figure 2:**
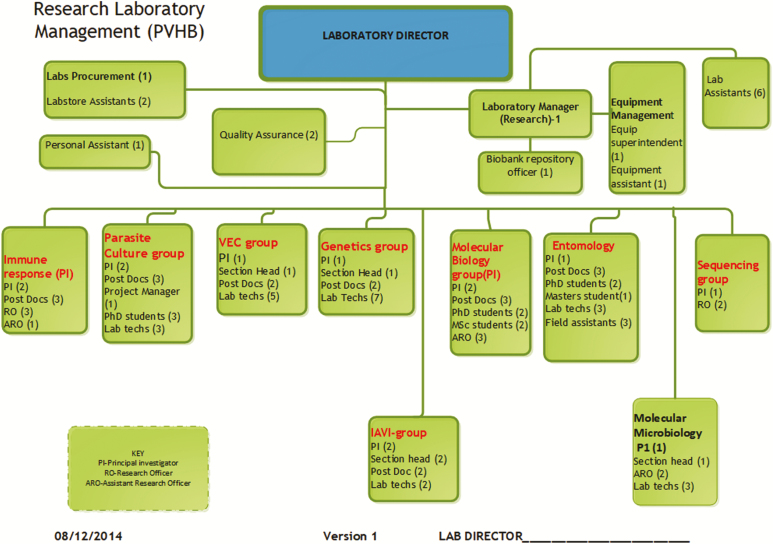
Immunology research laboratory organogram.

GCLP guidelines require that laboratory personnel should be qualified by education and experience, as well as have a personnel training file and formal reporting structures be in place to avoid conflict of interests.^1^ To achieve this, a staffing summary was performed and an organizational chart with reporting lines developed. This was done to meet the requirements of GCLP as well as the sponsors and to enhance communication and coordination, which, if not clearly addressed, can be a major detractor of quality system implementation, as indicated by Sollecito and Johnson.^2^ A quality assurance (QA) unit was formed to provide guidance and direction on the implementation of the GCLP standards. The organizational chart is described in **Figure 2**.

The laboratory director, who is charged with the overall responsibility of the GCLP compliance, was appointed and a research laboratory manager appointed to assist. The scientists, together with their research staff, were then grouped into their research clusters with the principal investigators (PIs) designated as the head of these groups. The QA unit had the responsibility of ensuring all protocols were approved, approving assay validation reports, developing standard operating procedures (SOPs), and conducting audits to determine compliance. The PIs were responsible for providing leadership and commitment within their research teams/groups. The staff within the research groups were tasked to write SOPs, validate assays, perform equipment management procedures, and develop their personnel training files.

All laboratory staff developed their personnel training files as required by the GCLP standards. Personnel training files contained records of formal qualification, professional qualification, resume, job descriptions, induction and orientation trainings, confidentiality statement, training and competency assessment records, registration with professional bodies, and immunization records. In addition to the above records, all staff were tasked to document trainings of various procedures relevant to their areas of work and competency assessments performed to ensure all staff were competent to perform the assigned procedures.^3^

## SOPs

GCLP guidelines require that all procedures be documented and written in a standard format. This is further elaborated in other standards, such as the Clinical and Laboratory Standards Institute and International Standards for Organization.^4^

Since research is an ever-evolving process aimed at discovering new concepts and enhancing scientific knowledge, most PhD students often keep changing the protocols and perform optimization on most assays to produce consistent results. Thus, it is difficult to develop and implement quality assurance system in a basic research laboratory.^5^ To establish an SOP system, the laboratory quality officer conducted an SOP training to all staff and developed an SOP template to be used to document all the procedures to ensure quality, integrity, and reliability of data generated.^3^ Each research team was then assigned SOPs to develop using the provided SOP template. The SOP template covered the following sections:

1. Introduction/purpose
2. Scope/responsibility
3. Safety precautions/risk assessments
4. Definitions and abbreviations
5. Specimen
6. Equipment/materials/reagents
7. Methodology
8. Related documents (eg, charts and forms)
9. References

This was only done to routine research procedures that had been optimized, validated, and approved by the laboratory director.^5^ All staff from each research group were then required to acknowledge these SOPs by reading and signing the SOPs’ attestation. The SOP review period was designed to be after every 2 years. The laboratory quality officer from the QA unit was responsible for coordinating all SOP revisions, distribution, removing obsolete copies from active use, and archiving. Any deviations from the SOPs were documented on a corrective action preventive action form and signed by the PI.

## Laboratory Analytical Plans

Even though the development of analytical plans as a component of a quality system is one of the items considered difficult to implement in a basic research laboratory, its implementation can be enhanced through flexible routines.

GCLP requires that all studies should have a documented laboratory analytical plans.^1^ The laboratory analytical plan is a formal document that reports all the study-related procedures within the laboratory. All the staff from the research groups were trained on writing a laboratory analytical plan by the laboratory quality officer, and an analytical plan template was developed to document all the existing and upcoming studies. The laboratory analytical plan template had the title of the study, list of procedures and their SOPs, roles of laboratories involved, a detailed list of the study’s organizational and management structure, description of laboratory procedures, confidentiality, and data analysis. The laboratory analytical plans helped in planning the conduct of the research studies and capturing some of the information about the laboratory that is not always indicated in the study protocol. All these laboratory analytical plans were finalized and approved by the laboratory quality officer and the PIs/study coordinators. Copies of approved laboratory analytical plans and protocols were filed in the laboratory for easy accessibility and reference.

## Assay Optimization and Validation

To produce accurate and reliable research data, these procedures need to be validated. Moreover, one of the GCLP requirements is to have all the research procedures validated. Validated and optimized procedures are easy to transfer from one laboratory to another, as suggested by Ozaki et al.^6^ To achieve this, numerous experiments were optimized to establish acceptance criteria for the formal validation experiments. Validation plans that include the selected parameters and a statistical analysis plan were developed and approved by the PIs.

## Equipment Management

GCLP guidelines require that the equipment used in the conduct of clinical research must be verified and “fit for purpose.”^1,4^ Documented evidence of proper installation, operation, maintenance, inspection, and calibration should be maintained to ensure test results are of high quality.^4^ Equipment assessment was performed to meet these requirements based on risk assessment. Moreover, an equipment inventory was developed to capture all the laboratory equipment available in the laboratory with information about the unique identification numbers of each piece of equipment, model/make, serial number, manufacturer, and location of the equipment.^7^ An equipment file with the required documentations was developed for each piece of equipment.

## Materials, Kits, and Reagents Management

All materials, kits, and reagents used within immunology were periodically checked to ensure that expired ones were removed from the laboratory. SOPs related to reagents preparation, labeling, and storage were developed and their records maintained by the laboratory quality officer. All reagents were labeled with the name of the reagent, their concentrations, date prepared, storage conditions, expiry date, and initials of the preparer. For those that did not have expiry dates indicated on them, their expiry dates were tracked from the date the reagents were opened. All these procedures ensured that reagents and kits remained stable to produce quality and reliable research data.

## Discussion

Setting up a quality assurance system using GCLP guidelines requires commitment from the management and the technical staff. The commitment demonstrated by the management and the staff was key to the successful implementation of the GCLP guidelines. Moreover, GCLP implementation also requires efficient coordination and communication since some of its aspects can be poorly accepted or misunderstood.^8^

GCLP implementation was successful at our laboratory because of three main reasons. First and most important was the efficient management structure that enhanced communication between the management and the staff. This structure also provided leadership and direction, making the staff develop interest in the implementation process. Second, the foundation of the laboratory best practices and quality culture was established by conducting technical and quality management system-related trainings, which made the implementation of the quality system a reality. The success was majorly attributed to the skilled and competent workforce who, after the trainings, used their knowledge and skills to implement the good laboratory practices, increase quality improvement, and resolve most of the findings raised by the laboratory quality officer. Conducting the trainings like SOP writing, good documentation practices, GCLP, improvement projects, and quality indicator trainings that targeted the implementation of various aspects of the quality system within the laboratory allowed more staff to be trained and helped encourage universal participation in building a competent and skilled workforce. Last, the application of common sense and good science knowledge were equally beneficial to achieve compliance.^9^

Despite the successful implementation of the GCLP process, some challenges were experienced. The biggest challenge was to adapt the GCLP guidelines to a basic research laboratory that did not conduct clinical trials. Thus, during implementation, we chose to implement the GCLP elements that could be applied to a basic research laboratory like SOPs, equipment management, laboratory analytical plans, and the organization and personnel. The other challenge was developing a deeper quality system that was simple, flexible, and able to add value to the organization considering good scientific and technical performance. The huge documentation that comes with the implementation of the GCLP guidelines needs to be thought of to ensure all staff can continue complying to GCLP guidelines for sustainability of the GCLP accreditation.

## Conclusions

The experience at KEMRI-Wellcome Trust Research Immunology basic laboratory during the GCLP implementation process clearly demonstrates what other laboratories that fully dedicate their concerted efforts can achieve in implementing a quality improvement process. Compliance with the GCLP standards, coupled with the periodic inspections, will ensure that the basic research performed meets the international standards of GCLP by producing valid and reliable data that can be reconstructed by other research organizations.
